# Gender-Specific Relationship between Obesity and Major Depression

**DOI:** 10.3389/fendo.2017.00292

**Published:** 2017-11-10

**Authors:** Li Li, Barbara A. Gower, Richard C. Shelton, Xiaoyan Wu

**Affiliations:** ^1^Department of Psychiatry and Behavioral Neurobiology, University of Alabama at Birmingham, Birmingham, AL, United States; ^2^Department of Nutrition Sciences, University of Alabama at Birmingham, Birmingham, AL, United States; ^3^Department of Nephrology, Wuhan University, Wuhan, China

**Keywords:** gender specific, obesity, depression, inflammatory markers, leptin

## Abstract

**Objective:**

Prior research suggests a bidirectional relationship between obesity and major depressive disorder (MDD), but the results have been heterogeneous. Differences between males and females in the association of MDD with obesity may contribute to inconsistent results. Thus, this study was designed to determine whether sex has a differential effect on the relationship between MDD and obesity, and to explore the potential mechanisms.

**Methods:**

All participants were diagnosed with MDD, and depression severity was measured using the 17-item Hamilton Depression Rating Scale. Body weight and height were measured to calculate body mass index (BMI). Body composition, including total fat, trunk fat, android fat, and visceral fat mass, was measured by dual-energy X-ray absorptiometry. Subjects provided blood samples, and serum was extracted for measuring the inflammatory factors using human immunoassay kits.

**Results:**

Among all obesity measures, depressed women had greater BMI and total body fat. By contrast, depressed men had greater visceral fat mass. However, only in depressed women was depression correlated with several measures of obesity, including BMI, total body fat, and visceral fat mass. A stepwise multiple regression analysis was conducted, and only visceral fat entered the regression model and was most predictive of depression in women (β = 0.60, *p* = 0.007). Moreover, compared with depressed men, depressed women had higher leptin levels after controlling for BMI, total body fat, and visceral fat.

**Conclusion:**

These results highlight gender differences in determining the association between obesity and depression, and elevated leptin level is a potential mechanism linking MDD to obesity in depressed women. Understanding a gender-specific relationship between obesity and MDD would allow clinicians to target and personalize therapies in the hope of improving health outcomes.

## Introduction

Major depressive disorder (MDD) and obesity are widely spread problems with high prevalence that are medically and economically costly to individuals and society. The prevalence of obesity among adults in the US is 32% (1), while that of MDD is 6.7% (2). Epidemiologic studies and clinical observations have indicated a possible overall association between MDD and obesity (3, 4). The association between depression and obesity was also reported in animal studies (5). However, these studies have yielded inconsistent findings, including evidence for no associations and negative associations (6, 7). Therefore, a definite conclusion cannot be established from prior findings. There are several possible explanations for these heterogeneous results. First, overweight and obesity were not differentiated in some studies although obesity is a more severe condition and more strongly associated with biological dysregulation and unfavorable health outcomes (1). Second, different measures of obesity have been applied, including body mass index (BMI), waist circumference, dual-energy X-ray absorptiometry, underwater weighing, and others. Third, ascertainment methods for MDD diagnosis and assessment have varied. Finally, the discrepancies may relate in part to moderators, including sex, age, and race that may vary between studies (8). In light of these, further studies are warranted to clarify the relationship between obesity and MDD, and possible moderators.

Epidemiologic data suggest a different prevalence of obesity in women and men with a greater prevalence in women than in men (1). Females are twice as likely as males to experience MDD (9, 10). Some previous reports also suggest that women respond differently than men to antidepressant treatment (11, 12). In particular, studies have noted a better response to selective serotonin reuptake inhibitor antidepressants in women and a better response to tricyclic antidepressants in men (11, 12). Although the sex-differential prevalence of obesity and depression as well as differential responses to antidepressants have been observed, an understanding of the sex differences in pathophysiology for these two common diseases is still very limited, which could be related to the lack of sex-specific data. Moreover, the lack of inclusion of sex-specific data collection also limits the ability to analyze sex differences in the outcomes of studies. The recognition of sex differences in the pathophysiology and the expression of human disease, including MDD and obesity, led to the NIH mandate to include both women and men in clinical studies and trials, and to analyze data by sex.

A number of studies have demonstrated that inflammation may contribute to the relationship between MDD and obesity. Systemic inflammation tends to increase as fat mass escalates, especially abdominal fat (13–18), and declines with fat loss (16, 18). Evidence from animal models and human studies indicates that inflammatory factors may contribute to the pathophysiology of MDD (19, 20). Cross-sectional studies have shown that many individuals with obesity and/or MDD have elevated levels of cytokines, including interleukin (IL)-6, tumor necrosis factor (TNF)-α and C-reactive protein (CRP) (13–16, 18–20). In addition, adipokines, including leptin and adiponectin, are shown to be involved in linking depression to obesity (21). Since both MDD and obesity are associated with a chronic inflammatory state, it is possible that the increased systemic inflammation may be a common pathway linking MDD with obesity.

Due to the increasing prevalence of obesity and MDD, and the uncertainty about possible sex differences in the relationship, we investigated the following research questions: (1) Is obesity associated with MDD in a sex-specific manner? (2) Are inflammatory markers involved in the sex-dependent associations between obesity and MDD? We hypothesize that MDD and obesity are associated, but that this association is in a sex-dependent manner, and that women may show stronger relationships between obesity and depression. A better understanding of the differences in obesity and MDD between the sexes, and translation of these differences into clinical practice could significantly improve treatment outcomes and address health disparities for women and men.

## Materials and Methods

### Subject Characteristics

Participants were screened and recruited from the psychiatric outpatient and inpatient units at the University of Alabama at Birmingham (UAB), or from the local community, Alabama. Participants were males and females aged 19–55 years and were physically healthy or had stable medical conditions. Race, education level, employment status, cigarette smoking, medical conditions, and medications were determined by self-report. All subjects were diagnosed with MDD according to the Diagnostic and Statistical Manual of Mental Disorders, fourth edition (DSM-IV), and confirmed *via* the Mini-International Neuropsychiatric Interview diagnostic scale (22). The samples were divided into two groups based on self-reported sex (females vs. males groups). The severity of depressive symptoms was determined using the 17-item Hamilton Depression Rating Scale (HAM-D) (23). Protocols were approved by the institutional review board at the UAB, and written informed consent was obtained from all subjects. Participants were excluded if they: (1) were taking medications known to affect weight, including antipsychotics and mood stabilizers; (2) were taking corticosteroids, antibiotics or anti-inflammatory medications; (3) had current infectious diseases or a history of autoimmune, endocrine, inflammatory disorders; (4) were pregnant or lactating; or (5) had a history of psychosis, bipolar disorders, illicit drugs or alcohol abuse.

### Measures of Obesity

Body weight and height were measured to calculate BMI using the Quetelet index (kg/m^2^). Body composition was determined in all subjects by the dual-energy X-ray absorptiometry (Lunar DXA, GE-Healthcare Madison, WI, USA) scanning. Participants were required to wear light clothes, remove all metal objects from their body, and lie supine with arms at their sides while they were completing the scan. The scanner allows the simultaneous measurement of total body fat, trunk fat and android fat mass independent of bone mass. CoreScan software was used to estimate the visceral fat mass based on measurement of abdominal area and subcutaneous adipose tissue (15, 24, 25).

### Serum Measures

Blood sample was collected from each participant and was centrifuged at 1,000 *g* for 15 min, immediately frozen at −80°C until analysis. The analysis for inflammatory markers, including interferon-γ (IFNγ), IL-6, IL-8, IL-10, and TNFα, was performed from serum samples using a Meso Scale Discovery multiplex assay (Gaithersburg, MD, USA), and were expressed in picograms per milliliter. CRP was analyzed using immunoassay on a Stanbio Sirrus Analyzer (Stanbio Laboratory, Boerne, TX, USA) using a Pointe Scientific (Canton, MI, USA) turbidometric reagent. Concentrations were expressed in milligrams per liter. Concentrations of leptin and adiponectin were also assayed and expressed in nanograms per milliliter and micrograms per milliliter, respectively, using commercially available radioimmunoassay kits according to the procedures supplied by the manufacturer (Millipore Corp., Billerica, MA, USA). All samples were assayed in duplicate, and the mean of each sample was reported.

### Statistical Analysis

All data are presented as mean ± SE unless otherwise stated. All statistical analyses were performed using the Statistical Package for the Social Sciences version 23 (SPSS Inc., IL, USA), and significant differences were set at *p* < 0.05. Normal distribution and homogeneity of variances were confirmed by Shapiro–Wilks *W* and Levene’s tests, respectively. Before analyses, CRP, TNFα, IFNγ, IL-6, IL-8, IL-10, leptin, and adiponectin were log transformed so that each of these variables followed an approximate normal distribution. Differences of variables in demographic and socioeconomic status, comorbidities and medications between the two groups were compared using independent *t*-test for continuous data and chi-square test for categorical data. Analysis of covariance (ANCOVA) was used to compare variables of obesity measures between depressed females and males after adjusting for age, race, and medications. Further analyses were conducted using ANCOVA to determine group differences in the inflammatory markers after controlling for age, race, medications, BMI, total body mass, and visceral fat mass. Relationships among depression severity and different measures of obesity in each group were examined using the partial correlation analysis. Multiple linear regression analysis was used to determine independent effects of variables of interest on depression in women and men, respectively. Stepwise multiple linear regression analysis was used to identify variables that best predicted depression.

## Results

### Characteristics of Study Subjects

Demographic and socioeconomic characteristics as well as comorbidities and current medications of study subjects are presented in Table 1. There were no significant differences in age, race, education levels, employment rate, and smoking status in females compared with males. Comorbidities, including hypertension and hyperlipidemia, and medications did not differ between the two groups.

**Table 1:** Demographic and socioeconomic characteristics, comorbidities, and medications in participants.

Variables	Males (*N* = 31)	Females (*N* = 36)	*p*-Values
Age (years, mean ± SE)	44.8 ± 2.3	44.2 ± 1.8	0.83
Caucasian/African American, *n*	18/13	17/19	0.46
Level of education (≥12 years), *n*	24	27	0.77
Currently employed, *n*	10	13	0.97
Smoking, *n*	10	12	0.59
**Comorbidities**			
Hypertension, *n*	9	14	0.61
Hyperlipidemia, *n*	3	5	0.72
**Medication**			
Hypertension, *n*	8	13	0.30
Hyperlipidemia, *n*	2	3	0.97
Antidepressants, *n*	22	32	0.12
Depressive score (mean ± SE)	21 ± 2	23 ± 2	0.66

### Relationships between MDD and Obesity

Comparing the HAM-D scores, women displayed similar depression severity compared with men (Table 1). Depressed women had greater BMI, total fat mass, but lower visceral fat mass compared with depressed men as listed in Table 2. Correlations were analyzed between the severity of depressive symptoms and obesity measures in both women and men. In depressed women, depression severity was positively associated with BMI (*r* = 0.35, *p* = 0.03), total body fat (*r* = 0.32, *p* = 0.045), and visceral fat mass (*r* = 0.52, *p* = 0.004). However, the same correlations were not statistically significant in depressed men. All associations between depression and obesity measures in women remained significant after controlling for age, race, and medications.

**Table 2:** Obesity measures in study subjects by gender.

Variables	Males	Females	*p*-Values
Weight (kg)	89.8 ± 3.5	87.8 ± 3.5	0.68
Body mass index (kg/m^2^)	28.2 ± 1.1	32.1 ± 1.2	**0.02**
Total fat (kg)	27.8 ± 2.4	37.6 ± 2.3	**0.004**
Trunk fat (kg)	16.0 ± 1.6	20.0 ± 1.4	0.06
Android fat (kg)	2.9 ± 0.3	3.6 ± 0.3	0.10
Visceral fat (kg)	1.5 ± 0.2	1.0 ± 0.1	**0.04**

*Data were presented as mean ± SE*.

*p values <0.05 are in bold font*.

### Inflammatory Markers in Patients with MDD

We next tested if there were differences in the levels of inflammatory markers between depressed women and men. There were no significant differences in the levels of CRP, IFNγ, IL-8, IL-10, TNFα, and adiponectin. By contrast, both IL-6 and leptin levels were significantly greater in depressed women compared with depressed men. However, the difference remained significant for leptin only after controlling for age, race, medications, BMI, total fat mass, and visceral fat mass (Table 3).

**Table 3:** Comparisons of inflammatory markers.

Variables	Males	Females	*P*_1_ value	*P*_2_ value^a^
CRP	3.7 ± 0.9	5.3 ± 1.0	0.21	0.30
IFNγ	3.64 ± 0.8	4.45 ± 0.9	0.54	0.40
IL-6	1.28 ± 0.3	1.23 ± 0.2	**0.04**	0.42
IL-8	9.7 ± 1.3	8.6 ± 0.6	0.93	0.87
IL-10	0.61 ± 0.12	2.82 ± 1.4	0.10	0.20
TNFα	4.7 ± 1.9	2.6 ± 0.2	0.66	0.46
Leptin	13.7 ± 2.1	40.7 ± 3.6	**<0.001**	**0.001**
Adiponectin	9.0 ± 1.1	9.8 ± 1.3	0.87	0.85

*CRP, C-reactive protein; IFN, interferon; IL, interleukin; TNF, tumor necrosis factor*.

*^a^P_2_ value is adjusted by body mass index, total fat, and visceral fat mass*.

*Data were presented as mean ± SE*.

*p values <0.05 are in bold font*.

### Prediction for Depression

To test for the independent contributions of each variable to depression, including race, age, employment, smoking, BMI, total fat, visceral fat mass, and leptin, multiple regression analyses were conducted in depressed women and men, respectively. The omnibus test of the model was significant in depressed women [*F*(8,28) = 2.97, *p* = 0.02], but not in depressed men [*F*(8,23) = 0.49, *p* = 0.84]. To identify which of these eight variables was most predictive of depression in women, a stepwise multiple linear regression analysis was performed. Analysis revealed that only visceral fat mass entered the regression equation with statistical significance and exerted an independent effect. The model could explain 34% of the variance in depression in women, and visceral fat mass contributed significantly (β = 0.60, *p* = 0.007).

## Discussion

Women are much more likely to experience depression than men, and epidemiologic data also suggest a higher prevalence of obesity in women than in men (1, 2, 9, 10). Due to this sex-differential higher prevalence of MDD and obesity in women and inconsistent reports regarding the association between MDD and obesity (3, 4, 6, 7), we tested the hypothesis that MDD is associated with obesity, but in a sex-specific manner. As hypothesized, we found a significant positive correlation between depression and the measures of obesity, including BMI, total body fat, and visceral fat mass, in females but not in males. In addition, multiple regression analyses revealed that depression might be able to be predicted by visceral fat mass in women, but not in men. Our findings indicate that previously observed relationships between obesity and depression may be true in women only.

A meta-analysis using community-based studies reported that depression was positively associated with obesity in the general population but appeared to be a gender-differential manner (26). Another study also showed that depression was strongly and consistently associated with obesity in women (27). Consistent with these previous findings, our study indicates a significant association between depression and obesity, however, it appears to be more prominent among women. Our results highlight sex differences in the determination of how depression is related to obesity.

In women, depression was positively correlated with several measures of obesity, including BMI, total body fat, and visceral fat mass. By contrast, depression was not related to any of the obesity measures in men in our study. It is interesting to note that no differences were seen in terms of depression severity between women and men. However, comparisons of obesity measures between women and men revealed that depressed women had a substantial greater BMI and total body fat, but less visceral fat mass. Our observations in patients with MDD are consistent with prior reports. Previous results showed that women had less visceral adipose tissue even though they had higher BMI, total body fat, and abdominal subcutaneous adipose tissue, compared with men (18). Combined with our results, this may hold true regardless of the depressive status. It is speculative that women with MDD may have the shuttling of fat to abdominal subcutaneous adipose tissue or other compartment. Indeed, in recent years, abdominal subcutaneous adipose tissue is believed to confer increased metabolic and cardiovascular risks (28–30). Given this, further studies are warranted to directly investigate the roles of subcutaneous adipose tissue in obesity-related disorders in the depressed patients, women in particular.

Multiple regression analyses revealed that obesity measures along with other parameters could predict depression in women, but only visceral fat was the most predictive of depression. These findings emphasize that visceral fat may have a stronger relationship with depression. Consistently, increasing evidence has indicated that the proportion of abdominal adipose tissue, visceral adipose tissue in particular, is a key correlate and perhaps a main factor of obesity-related health problems because it has been associated with an increased risk of comorbidities such as type 2 diabetes, stroke, sleep apnea, hypertension, dyslipidemia, and some types of cancer (31–33). In line with our previous and others findings, this study highlights the importance of visceral fat as a marker of obesity-related disorders, including depression, particularly in women.

The mechanisms that women and men exhibit different relationships between depression and obesity are unknown. One possible explanation is that this sex-differential association between obesity and MDD may highlight sex differences in biological vulnerability as well as may be directly related to sex hormones, which are reported to be linked with depressed mood (34, 35). Another explanation for a sex-specific association between obesity and MDD may arise from the differences in the inflammatory process. Our findings indicate that female patients with MDD have elevated circulating leptin levels compared with depressed males. Leptin, an adipocyte-derived protein, has emerged as a key player in the pro-inflammatory process in obesity and metabolic syndrome and is, therefore, considered a pivotal link in obesity-related diseases (36, 37). Prior literature has also suggested a relationship between leptin and depression (19, 20, 38, 39). A large longitudinal study demonstrated that serum leptin concentrations predicted the later development of major depression (40). Moreover, some studies focusing on leptin and depression showed sex differences in the relationship. Esel group reported a positive correlation between leptin levels and depression symptoms in women only (41). Consistently, greater leptin levels were found in depressed women, but not men, when compared with controls in other studies (42). One recent study showed that reduced leptin concentrations could be a potential predictor of reduced depression symptoms in obese adolescents, and the results were stronger in girls than in boys (43). In agreement with these findings, we found that depressive patients overall had elevated serum leptin concentrations compared with controls (data not shown), but elevated circulating leptin levels were more prominent in depressed women than in depressed men after controlling for covariates including age, race, and obesity measures. Although it is as yet unclear why there is a gender difference in circulating leptin levels in depression, our study may have uncovered a potential biological link between circulating leptin levels and depression, particularly in women, in the presence of abdominal obesity.

By contrast, we did not find elevated IL-6 in depressed women compared with depressed men after controlling for obesity measures. Increasing evidence has indicated a role of elevated IL-6 in the pathogenesis of MDD, however, the majority of studies were correlational and cross-sectional with high heterogeneity among studies. A recent meta-analysis using longitudinal studies showed that the weighted-mean effect size for IL-6 is relatively small (44). Indeed, our finding in terms of IL-6 is supported by prior two studies from our group. One study did not identify a significant elevation of IL-6 in depressed women compared with depressed men (45). The other suggested that higher IL-6 in MDD patients may be explained, at least in part, by obesity (20). Taken together, the role of elevated IL-6 levels in depressed patients may need to be interpreted in a sex-specific manner as well as to include other confounding factors such as obesity. Thus, more rigorous and longitudinal studies in humans are needed to establish the sex-IL-6 relationship in patients with MDD and/or obesity.

Various instruments are available to assess the severity of depressive symptoms in MDD, ranging from self-reported to clinicians-managed. One possible explanation for the heterogeneous reports regarding the relationship between obesity and MDD in the previous studies might be due to methodological variations to diagnose and assess MDD. In this study, DSM-IV diagnostic criteria for MDD were applied on the basis of data from a structured psychiatric interview, and HAM-D was used by the clinicians, which could avoid the inaccurate self-reporting caused by social desirability and difficulties in self-observation. This could hold true especially in men, leading to methodologic variations and different outcomes.

The results of this study may need to be interpreted with caution in light of several limitations. In spite of significant associations between depression and obesity in females, a limited sample size is a primary limitation of our study, especially for interpretations of depression and obesity in males. Thus replication and expansion of our results in a larger sample would enhance the generalizability of our findings. Second, a cross-sectional study design did not allow us to examine for causal inferences regarding the development of obesity and MDD or their relationships over time. Future work is therefore required to focus on prospective studies to establish a causal relationship between obesity and MDD, especially in women, for directional effects as well as potential implications for the depression–obesity comorbidity. Majority of the MDD participants who completed the study were taking antidepressants; however, none of them were taking antipsychotics, mood stabilizers, or any other medications like corticosteroids that are known to affect body weight. Although medications, including antidepressants, were controlled in our analyses, future studies assessing the impact of antidepressants on the relationship between MDD and obesity would have been a valuable addition.

In summary, we observed an association between obesity and MDD, in particular, a correlation between depression and measures of obesity, including BMI, total body fat, android fat, and visceral fat mass, in females only. Our findings corroborate and extend previous findings, and provide further evidence for a sex-specific relationship between obesity and MDD. The differential impact of gender on the relationship between MDD and obesity is particularly of interest in the treatment of the obesity and depression in women. Further research employing longitudinal human studies as well as experimental interventions is needed to elucidate cause and effect relationships, and to provide sex-specific preventive and therapeutic strategies for women with MDD and obesity.

## Ethics Statement

Protocols were approved by the institutional review board at the University of Alabama at Birmingham, and written informed consent was obtained from all subjects.

## Author Contributions

LL designed the experiments, analyzed and interpreted the data, and wrote the manuscript. RS contributed to experimental design and reviewed/edited the manuscript. BG contributed to experimental design, data interpretation, and reviewed/edited the manuscript. XW contributed to review and edit the manuscript. All the authors approved the submission and publication.

## Conflict of Interest Statement

The authors declare that the research was conducted in the absence of any commercial or financial relationships that could be construed as a potential conflict of interest.
